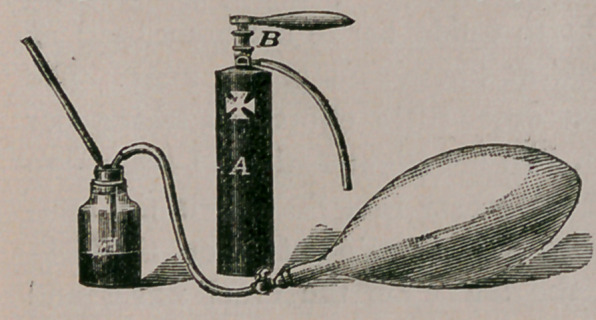# Oxygen—Its Agency in Therapeutics

**Published:** 1887-01

**Authors:** B. M. Lawrence


### OXYGEN, ITS AGENCY IN THERAPEUTICS.

BY B. M. LAWRENCE, M. D.

No. 2.

For years past there has been a growing tendency on the part of physicians to substitute more natural and harmless remedial agents for the dangerous heroic treatment formerly held in such great favor, and the deadly drugs are daily loosing caste. Powerful active poisons, if administered at all, are more usually employed in small or homæpathetic doses, and the finer forces of sunlight, electricity, massage and magnetism are rapidly becoming recognized agents in modern therapeutics, and no little interest is now being manifested in impenetrable, mental and physiological impressions as important factors in healing the sick, faith or prayer cures, which are frequently reported as having produced marvelous results in restoring invalids. Occupying the middle ground between the subtile and intangible forces on the one hand, and the grosser material on the other, there is no curative agent at present which is attracting more general attention than that of oxygen. This most abundant, active and powerful element was unknown to the ancients, having been discovered a little more than one hundred years since by Dr. Priestley, and about the same time by Dr. Scheele. It was demonstrated that birds and animals, when caused to inhale the gas, soon became intensely exhilarated. That when dogs breathed oxygen before the chase they not only became more lively, active and animated, but they also became able to run farther, faster, and endure a greater physical strain for a much longer period of time, showing a decided increase of muscular strength ; and that animals when compelled to inhale noxious gases until they become asphyxiated and nearly lifeless, would revive almost immediately when treated with pure oxygen gas.

Savosier, by frequently repeating these experiments, discovered that the muscles of animals daily subjected to the influence of *vital air*—the name first given to oxygen gas—soon became tough, firm and hard, indicating greater strength and endurance. The close relation well-known to exist between physical strength and perfect health, immediately suggested to the minds of the early experimenters with oxygen, that it might become a valuable agent in therapeutics ; that the gas would give tone, vigor, strength and vitality to the sick and feeble as well as to animals, and thus become a pleasant and popular method of curing disease.

Dr. Beddoes, a professor of chemistry at Oxford in 1789, was the first among the eminent medical men of his time who gave the subject a careful and scientific investigation. Sir Humphrey Davy in connection with other leading scientists also became greatly interested in the subject, and ably assisted in testing the action of oxygen as a curative agent. But one great disadvantage under which they conducted their researches was the difficulty of preparing sufficient quantities of oxygen gas of a quality pure enough to be used for medical purposes.

Dr. Beddoes finally became discouraged on account of the many accidents and obstacles he was compelled to encounter, including the torrent of ridicule poured upon him continually by a class of physicians and conservatives who always oppose every innovation ; but he never once lost faith in the new remedy, although compelled to admit his inability to render it available in practice. Drs. Hill, Thornton, Cavallo, McCormack and others were sufficiently encouraged by the degree of success that had attended the efforts of their predecessors, to follow up the subject with more extended experiments, which they conducted as far as possible in comparative seclusion, in order to escape professional criticism, and although they failed to attract the attention of medical men, yet they each most emphatically expressed their conviction that oxygen would eventually be acknowledged as one of the best and most efficient among the potent and valuable agencies in therapeutics.

Dr. Hill, in 1820, published a brochure filled with highly instructive facts and suggestions respecting the nature of their investigations, and the effect of inhaling an artificial or super-oxygenated atmosphere in a large class of diseases, including some very obstinate cases of *asthma, pulmonary and cardiac weakness, careis, scrofula, spinal deformities, hip-joint disease, arested development, malignant tumors, ulcers, lupus, intractable skin diseases, neuralgia, hysteria, and various forms of female disorders*, and in every case the result was more or less encouraging ; and in some instances truly remarkable. The report of these highly successful experiments failed, however, to attract any very general attention to the subject, and not until the last two or three decades has the inhalation of oxygen as an adjunct in the treatment of disease begun to claim the attention of the mass of medical men ; literature on the subject is still limited, and although little has been written—at least until quite recently—yet we find such eminent men as Liebig, Goolden, Birch, Demarquay, Richardson and many others have been more or less interested investigators, using it frequently in their practice, and without exception all admitting the potency of oxygen as a natural curative. The medical journals and writers at home and abroad have of late, however, been directing public attention to the use of super-oxygenated air as a safe, pleasant and powerful restorative.

"Pneuma Therapuea" is the title of a small anonymous volume published in London, in 1856. It contained a report of cases treated, repeated some of the experiments made by Dr. Hill, including the treatment of *dyspepsia, chronic dysentery, dyspnœa and asphyxia*, with a reference to the investigations of Beddoes, Davy, Werks, Manzies, Liebig and others. The appendix advertised to supply both oxygen and apparatus for its manufacture and inhalation.

Dr. Birch, of the Manchester Medical School, in 1857 published a monograph "On Oxygen," the second edition of which was perhaps the first literature on the subject which commanded any special attention. The author, on page 148, says of artificially prepared oxygen: "It is a powerful, really scientific and agreeable curative agent, and is capable of a far more extensive range in its application to the rational treatment of chronic disease than perphaps any other remedy, *is pre-eminently Nature's therapeutic*, affording assistance in her own way, without opposing the intentions of her ever present *vis medicatrix*, and is entitled to the position of a curative in a variety of intractable diseases, otherwise incurable by any known means, * * * * and is occasionally *the* remedy, and then the only one worthy of the name, in certain contingencies where life must be and frequently is sacrificed by neglecting a fair trial of it ;" and he adds: "It may safely be predicted that sooner or later non-atmospheric oxygen will be universally admitted as one of our most valued remedial agents."

Prof. Da Costa, "On Inhalations," published in 1867, says: "Of all the gases, oxygen is now being most tried practically for the relief of dyspnea, and in low fevers, * * * * and in chlerosis. Excellent results have quite lately been obtained for oxygen by Demarquay, who is at present investigating the subject, not only in the conditions named, but in diabetes, in sinile gangrene, and in prolonged suppurations."

Dr. S. S. Wollian, one of the earliest and ablest advocates of "super-Oxygenation as a Therapeutic Measure" in this country, published a series of articles in the Chicago *Medical Journal* during the year 1869, giving the results of his experience with oxygen and its compounds in the treatment of a great variety of nervous and chronic ailments, and since that time he has kept the subject before the profession by his contributions to the *Medical Record* and other periodicals "On Oxygen as a Remedial Agent," page 11, he says: "The past decade in the history of medicine may not inaptly be termed *the germ craze.* The microscope has monopolized the laboratory, and well-nigh superseded the inductive method * * * * The shortcomings of impure or carelessly manufactured gas have done more than all else to bring the use of oxygen and its congeners into disregard, if not into discredit, and to prevent its timely use by the profession at large."

Referring to the difficulty of generating or procuring a *pure gas* as one of the "drawbacks to its general use by even those who are aware of its value, and anxious to avail themselves of it," he says: "Some apparatus is required, and careful chemical manipulations, which in time no doubt will be delegated to the pharmacist and manufacturing chemist." That time has already come since the above was written, and "the apparatus neither complicated nor expensive" has been devised whereby the best and purest oxygen compounds are compressed into strong wrought iron cylinders, making it perfectly portable, convenient and economical for the patient at home or at the physician's office.

Dr. J. P. Turner, one of the pioneers in practically employing oxygen and its compounds for the cure of disease during the past twenty-five years—with his associate, including the writer—was the first to prepare and use the liquified gas, which he condensed with powerful steam force-pumps under the pressure of more than sixty atmospheres. He and his associates have probably had a larger and more successful experience with the inhalation of oxygen, ozone and other remedies than any of the many physicians who are interested in this comparatively unexplored field of the healing art. Some clinical experience and caution with regard to impositions palmed off as "compound oxygen" by the unscrupulous pretenders, whose greed of gain would trifle with the lives of those who are almost hopeless invalids, will form a leading feature of the next article for the JOURNAL.

## Figures and Tables

**Figure f1:**